# Synthesis of the tetrasaccharide repeating unit of the *O*-specific polysaccharide of *Azospirillum doebereinerae* type strain GSF71^T^ using linear and one-pot iterative glycosylations

**DOI:** 10.3762/bjoc.16.141

**Published:** 2020-07-15

**Authors:** Arin Gucchait, Pradip Shit, Anup Kumar Misra

**Affiliations:** 1 Bose Institute, Division of Molecular Medicine, P-1/12, C.I.T. Scheme VII M, Kolkata 700054, India

**Keywords:** *Azospirillum doebereinerae*, glycosylation, HClO_4_-SiO_2_, *O*-polysaccharide, tetrasaccharide

## Abstract

A straightforward synthetic strategy was developed for the synthesis of the tetrasaccharide repeating unit corresponding to the *O*-specific polysaccharide of *Azospirillum doebereinerae* type strain GSF71^T^ in a very good yield adopting sequential glycosylation followed by removal of the *p*-methoxybenzyl (PMB) group in the same pot. Further, the synthetic strategy was modified by carrying out three stereoselective iterative glycosylations followed by in situ removal of the PMB group in one pot. The stereochemical outcome of the newly formed glycosidic linkages was excellent using thioglycoside derivatives as glycosyl donors and a combination of *N*-iodosuccinimide (NIS) and perchloric acid supported on silica (HClO_4_-SiO_2_) as the glycosyl activator.

## Introduction

The development of plant-growth-promoting agents has become an attractive area of research in the agricultural sciences to reduce the need for chemical fertilizers, which cause environmental pollution, resistance in plant pathogens as well as high production costs of crops [1–3]. Varieties of crop plants have symbiotic relationships with microorganisms by hosting them in their roots and utilize their beneficial effects such as improving the quality of soil and environment by nitrogen fixation [4]. *Azospirillum* bacteria are Gram-negative proteobacteria mostly found in the rhizosphere of wild and cultivated grasses and crops [5–7]. There are several species of *Azospirillum*, which were found to act as potent plant-growth stimulators [8]. In addition to the nitrogen fixing capabilities, *Azospirillum* produce a number of plant-growth-promoting vitamins and phytohormones [9–10]. Their potential to promote resistance towards bacterial infections in rice was also documented [11]. The potential of stimulating plant-growth-related biochemical processes of *Azospirillum* rely on capsular and *O*-specific polysaccharides present in the bacteria cell wall [12]. Due to their structural orientation, cell-wall polysaccharides play a pivotal role in the initial stage of the host–microbes interactions and colonization of rhizobacteria in the plant roots [13]. Therefore, it is quite pertinent to study the effect of the cell-wall polysaccharides of rhizobacteria on the growth of leguminous plants. As a consequence, the structural elucidation of a variety of cell-wall polysaccharides of several strains of *Azospirillum* have been reported in the past [14–19]. However, detailed biological studies to understand the biochemical role of cell-wall polysaccharides require significant quantities of the polysaccharides with adequate purity, which are difficult to achieve through isolation from the natural sources. Therefore, the development of chemical synthetic strategies could provide access to large quantities of the oligosaccharides with defined chemical structures and free from biological impurities. Among the nineteen species of *Azospirillum*, *Azospirillum doebereinerae* was isolated from the rhizosphere of the biomass producing plant, *Miscanthus giganteus* [20]. Recently, Sigida and co-workers reported the structure of the tetrasaccharide repeating unit of the *O*-specific polysaccharide of *Azospirillum doebereinerae* type strain GSF71^T^, which is composed of three α-ʟ-rhamnose units and one β-ᴅ-glucosamine moiety [21]. It also contains two *O*-acetyl groups which might play a significant role in the biological activities of the polysaccharide. In this context, a straightforward synthesis of a tetrasaccharide corresponding to the cell-wall *O*-polysaccharide of the *Azospirillum doebereinerae* type strain GSF71^T^ using a sequential as well as three-step iterative glycosylation in one pot was developed and is reported herein.

## Results and Discussion

**Figure 1:**
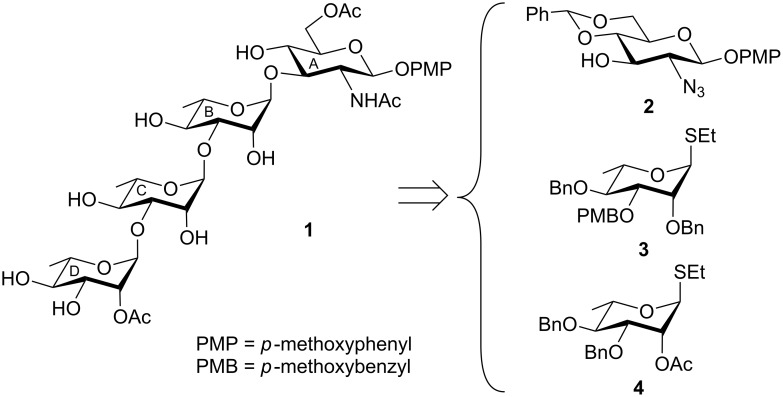
Structure of the synthesized tetrasaccharide and its synthetic intermediates.

At the beginning, the retrosynthetic analysis suggested that a sequential glycosylation reaction using judiciously protected monosaccharide thioglycosides could be the best strategy for achieving the desired tetrasaccharide **1**. Accordingly, differentially protected monosaccharide intermediates such as ᴅ-glucosamine derivative **2** [22], ʟ-rhamnose derivatives **3** [23], and **4** [24] were prepared following the literature reported reaction conditions. The selection of the *p*-methoxybenzyl (PMB) group for the temporary protection of the chain elongation C-3 hydroxy group in the ʟ-rhamnosyl thioglycoside donor **3** allowed carrying out the stereoselective glycosylation steps followed by the removal of the PMB group [25] in one pot. This was achieved by using a combination [26–30] of *N*-iodosuccinimide (NIS) and perchloric acid supported on silica (HClO_4_-SiO_2_) [31–32]. HClO_4_-SiO_2_ was used as a noncorrosive solid acid in the glycosylation reactions. The selection of functional groups and their post-glycosylation modifications led to the formation of partially *O*-acetylated tetrasaccharide **1**, as found in the naturally isolated repeating unit of the polysaccharide (Figure 1).

**Scheme 1:**
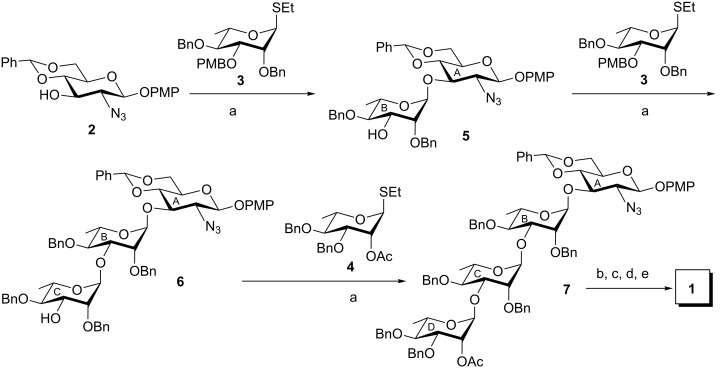
Stepwise stereoselective synthesis of tetrasaccharide **1**. Reagents and conditions: (a) NIS, HClO_4_-SiO_2_, CH_2_Cl_2_, MS 4 Å, −15 °C, 30 min, then 20 °C, 30 min, 73% for compound **5**, 70% for compound **6**, and 74% for compound **7**; (b) CH_3_COSH, pyridine, rt, 16 h; (c) HClO_4_-SiO_2_, CH_3_CN, rt, 15 min; (d) acetic anhydride, pyridine, CH_2_Cl_2_, 0 °C, 5 h; (e) Et_3_SiH, 20% Pd(OH)_2_/C, CH_3_OH, rt, 24 h, 50% overall yield for three steps.

The stereoselective glycosylation of compound **2** with ʟ-rhamnosyl thioglycoside **3** in the presence of a combination of NIS and HClO_4_-SiO_2_ [26–27] followed by removal of the PMB group [25] from the product in the same pot by tuning of the reaction conditions led to the formation of disaccharide acceptor **5** in 73% yield. The quantity of HClO_4_-SiO_2_ present in the reaction mixture was very low, which allowed the selective deprotection of the highly acid labile PMB group without affecting the benzylidene acetal in the molecule in dichloromethane as the solvent. The stereochemical outcome of the newly formed glycosidic linkage was confirmed by NMR spectroscopic analysis [appearance of signals at δ 5.47 (s, PhC*H*), 5.13 (br s, H-1_B_), 4.81 (d, *J* = 7.5 Hz, H-1_A_) in the ^1^H NMR and at δ 102.1 (C-1_A_), 102.0 (Ph*C*H), 97.8 (C-1_B_) in the ^13^C NMR spectrum, respectively]. Repeating the reaction with disaccharide acceptor **5** using the same conditions, the stereoselective glycosylation with compound **3** furnished trisaccharide acceptor **6** in 70% yield. The desired stereochemistry of the newly formed glycosidic bond was confirmed by NMR spectroscopic analysis [appearance of signals at δ 5.54 (s, PhC*H*), 5.17 (br s, H-1_C_), 5.11 (br s, H-1_B_), 4.82 (d, *J* = 8.0 Hz, H-1_A_) in the ^1^H NMR and at δ 102.1 (C-1_A_), 102.0 (Ph*C*H), 98.7 (C-1_C_), 98.4 (C-1_B_) in the ^13^C NMR spectrum, respectively]. Although the C-2 position of the ʟ-rhamnosyl donor **3** was protected with a nonparticipating benzyl group, all glycosylation reactions furnished exclusively 1,2-*trans*-glycosidic linkages under anomeric control. Subjecting compound **6** to the stereoselective glycosylation conditions using ʟ-rhamnosyl thioglycoside **4** afforded the tetrasaccharide derivative **7** in 74% yield. The formation of tetrasaccharide derivative **7** was supported by NMR spectral analysis [signals at δ 5.51 (s, PhC*H*), 5.12 (br s, H-1_D_), 5.08 (br s, H-1_C_), 5.07 (br s, H-1_B_), 4.80 (d, *J* = 8.0 Hz, H-1_A_) in the ^1^H NMR and at δ 102.1 (C-1_A_), 101.9 (Ph*C*H), 99.3 (2 C, C-1_B_, C-1_C_), 98.4 (C-1_D_) in the ^13^C NMR spectrum, respectively] (Scheme 1).

**Scheme 2:**
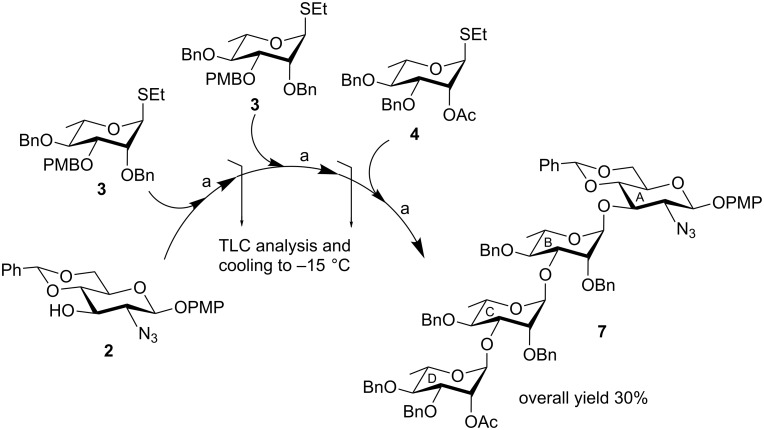
Iterative stereoselective three-step one-pot glycosylation. Reaction conditions: (a) NIS, HClO_4_-SiO_2_, CH_2_Cl_2_, MS 4 Å, –15 °C, 30 min, then 20 °C, 30 min.

Having obtained the tetrasaccharide derivative **7** in a very good yield in a minimum number of steps by the sequential synthetic strategy, we decided to explore the possibility of achieving compound **7** by carrying out three iterative glycosylation steps and in situ removal of the PMB groups from the glycosylation products in a one-pot reaction. Based on the findings of the sequential glycosylations, two consecutive NIS and HClO_4_-SiO_2_-mediated glycosylations were carried out starting with compound **2** and thioglycoside **3** followed by the removal of the PMB group from the intermediate products by changing the temperature of the reaction and coupling the in situ-generated trisaccharide derivative **6** with compound **4** to furnish the desired tetrasaccharide derivative **7** in 30% overall yield. The structure of compound **7** obtained by the three iterative glycosylation reactions in one pot fully matched with the product obtained following the sequential glycosylation strategy (Scheme 2). It is noteworthy that in parallel to the sequential glycosylation strategy, the desired tetrasaccharide derivative **7** could be achieved in comparably good yield following the one-pot protocol involving the three iterative glycosylations without the isolation and purification of the intermediate glycosylation products.

Finally, compound **7** was subjected to a set of reactions involving (a) transformation of the azido group to an acetamido group by the treatment with thioacetic acid [33]; (b) removal of the benzylidene acetal by the treatment with HClO_4_-SiO_2_ [34]; (c) selective acetylation of the primary hydroxy group, and (d) removal of the benzyl groups by catalytic transfer hydrogenation [35] using triethylsilane in the presence of Pearlman’s catalyst [36] to furnish the desired tetrasaccharide **1** in 50% overall yield with the two *O*-acetyl groups at the respective positions as found in the isolated *O*-specific polysaccharide. The formation of compound **1** was unambiguously confirmed by its NMR spectroscopic analysis [signals at δ 5.03 (br s, H-1_D_), 5.03 (d, *J* = 10.0 Hz, H-1_A_), 4.92 (br s, H-1_C_), 4.78 (br s, H-1_B_) in the ^1^H NMR and at δ 102.2 (C-1_C_), 101.3 (C-1_B_), 99.8 (C-1_A_), 99.3 (C-1_D_) in the ^13^C NMR spectrum, respectively] (Scheme 1).

## Conclusion

In summary, the tetrasaccharide repeating unit of the *O*-specific polysaccharide of *Azospirillum doebereinerae* type strain GSF71^T^ was synthesized in good yield using a sequential as well as a one-pot iterative glycosylation approach. The removal of the PMB group was achieved in situ in the same pot mediated by NIS and HClO_4_-SiO_2_. The glycosylation steps were high yielding with satisfactory stereochemical outcome. The application of the generalized glycosylation conditions using a common glycosyl donor provided the desired compound in a minimum number of reaction steps. HClO_4_-SiO_2_ was used as a solid acid activator in the glycosylation reactions as well as for functional group transformation.

## Experimental

**General methods:** All reactions were monitored by thin-layer chromatography using silica gel-coated TLC plates. The spots on TLC were visualized by warming ceric sulfate (2% Ce(SO_4_)_2_ in 2 N H_2_SO_4_) sprayed plates on a hot plate. Silica gel 230–400 mesh was used for column chromatography. NMR spectra were recorded on a Bruker Avance 500 MHz using CDCl_3_ as solvent and TMS as internal reference unless stated otherwise. Chemical shift values are expressed in δ ppm. The complete assignment of proton and carbon spectra was carried out by using a standard set of NMR experiments, e.g., ^1^H NMR, ^13^C NMR, ^13^C DEPT 135, 2D COSY, and 2D HSQC etc. HRMS were recorded on a Bruker mass spectrometer. Optical rotations were recorded in a Jasco P-2000 spectrometer. Commercially available grades of organic solvents of adequate purity were used in all reactions.

**Preparation of the HClO_4_-SiO_2_ catalyst:** The catalyst was prepared in a similar manner as described earlier [28,37]. In brief, HClO_4_ (1.8 g, 12.5 mmol, as a 70% aqueous solution) was added to a suspension of SiO_2_ (230–400 mesh, 24 g) in Et_2_O (70 mL). The mixture was concentrated and the residue was heated at 100 °C for 72 h under vacuum to furnish HClO_4_-SiO_2_ (0.5 mmol/g) as a free flowing powder. **Caution!** Although no explosions were reported under these conditions, special care should be taken for large-scale preparations.

***p*-Methoxyphenyl (2-*O*-acetyl-α-ʟ-rhamnopyranosyl)-(1→3)-(α-ʟ-rhamnopyranosyl)-(1→3)-(α-ʟ-rhamnopyranosyl)-(1→3)-2-acetamido-6-*O*-acetyl-2-deoxy-β-ᴅ-glucopyranoside (1):** To a solution of compound **7** (300 mg, 0.21 mmol) in pyridine (2 mL) was added CH_3_COSH (1 mL, 14.2 mmol) and the reaction mixture was stirred at room temperature for 12 h. The solvents were removed under reduced pressure and the product was passed through a short pad of SiO_2_ using hexane/EtOAc 2:1 to give the *N*-acetylated product. To a solution of the product in CH_3_CN (10 mL) was added HClO_4_-SiO_4_ (50 mg) and the mixture was allowed to stir at room temperature for 15 min. Then, the reaction mixture was filtered and the filtrate was concentrated under reduced pressure. To a solution of the diol in CH_2_Cl_2_ (5 mL) were added pyridine (100 μL, 1.24 mmol), acetic anhydride (40 μL, 0.42 mmol), and the reaction mixture was stirred at 0 °C for 5 h. The solvents were removed under reduced pressure and the product was passed through a short pad of SiO_2_ to give the selectively acetylated product. To a solution of the product in CH_3_OH (5 mL) were added 20% Pd(OH)_2_/C (100 mg) and Et_3_SiH (3 mL, 18.8 mmol) and the resulting mixture was allowed to stir at room temperature for 24 h. The reaction mixture was filtered through a Celite bed and washed with CH_3_OH (25 mL). The filtrate was concentrated under reduced pressure and passed through a Sephadex LH-20 column using CH_3_OH/H_2_O 6:1 as eluent to give pure compound **1** (90 mg, 50%).

***p*-Methoxyphenyl (2,4-di-*O*-benzyl-α-ʟ-rhamnopyranosyl)-(1→3)-2-azido-4,6-*O*-benzylidene-2-deoxy-β-ᴅ-glucopyranoside (5):** To a solution of compound **2** (600 mg, 1.5 mmol) and compound **3** (800 mg, 1.57 mmol) in anhydrous CH_2_Cl_2_ (10 mL) was added activated MS 4 Å (500 mg) and the mixture was cooled to −15 °C under Ar. To the cold reaction mixture was added NIS (360 mg, 1.6 mmol) followed by HClO_4_-SiO_2_ (40 mg), and the mixture was stirred at the same temperature for 30 min. After complete consumption of compound **3** [TLC; hexane/EtOAc 2:1], the reaction mixture was allowed to stir at 20 °C for 30 min, filtered and washed with CH_2_Cl_2_ (30 mL). The organic layer was successively washed with 5% Na_2_S_2_O_3_ (25 mL), satd. NaHCO_3_ (25 mL), and H_2_O (25 mL), dried (Na_2_SO_4_), and concentrated to give the crude product, which was purified on SiO_2_ using hexane/EtOAc 3:1 as eluent to give pure compound **5** (806 mg, 73%).

***p*-Methoxyphenyl (2,4-di-*O*-benzyl-α-ʟ-rhamnopyranosyl)-(1→3)-(2,4-di-*O*-benzyl-α-ʟ-rhamnopyranosyl)-(1→3)-2-azido-4,6-*O*-benzylidene-2-deoxy-β-ᴅ-glucopyranoside (6):** To a solution of compound **5** (700 mg, 0.96 mmol) and compound **3** (515 mg, 1.01 mmol) in anhydrous CH_2_Cl_2_ (10 mL) was added activated MS 4 Å (500 mg) and the mixture was cooled to −15 °C under Ar. To the cold reaction mixture was added NIS (230 mg, 1.02 mmol) followed by HClO_4_-SiO_2_ (30 mg) and it was stirred at the same temperature for 30 min. After complete consumption of compound **3** [TLC; hexane/EtOAc 1:1], the reaction mixture was allowed to stir at 20 °C for 30 min, filtered, and washed with CH_2_Cl_2_ (30 mL). The organic layer was successively washed with 5% Na_2_S_2_O_3_ (25 mL), satd. NaHCO_3_ (25 mL), and H_2_O (25 mL), dried (Na_2_SO_4_) and concentrated to give the crude product, which was purified on SiO_2_ using hexane/EtOAc 3:1 as eluent to give pure compound **6** (704 mg, 70%).

***p*-Methoxyphenyl (2-*O*-acetyl-3,4-di-*O*-benzyl-α-ʟ-rhamnopyranosyl)-(1→3)-(2,4-di-*O*-benzyl-α-ʟ-rhamnopyranosyl)-(1→3)-(2,4-di-*O*-benzyl-α-ʟ-rhamnopyranosyl)-(1→3)-2-azido-4,6-*O*-benzylidene-2-deoxy-β-ᴅ-glucopyranoside (7):** To a solution of compound **6** (600 mg, 0.57 mmol) and compound **4** (270 mg, 0.63 mmol) in anhydrous CH_2_Cl_2_ (10 mL) was added activated MS 4 Å (500 mg) and the mixture was cooled to −15 °C under Ar. To the cold reaction mixture was added NIS (150 mg, 0.67 mmol) followed by HClO_4_-SiO_2_ (20 mg) and it was stirred at same temperature for 30 min. The reaction mixture was filtered and washed with CH_2_Cl_2_ (30 mL). The organic layer was successively washed with 5% Na_2_S_2_O_3_ (25 mL), satd. NaHCO_3_ (25 mL), and H_2_O (25 mL), dried (Na_2_SO_4_) and concentrated to give the crude product, which was purified on SiO_2_ using hexane/EtOAc 2:1 as eluent to give pure compound **7** (596 mg, 74%).

**Using one-pot iterative glycosylations from compound 2:** To a solution of compound **2** (300 mg, 0.75 mmol) and compound **3** (390 mg, 0.77 mmol) in anhydrous CH_2_Cl_2_ (5 mL) was added activated MS 4 Å (200 mg) and the mixture was cooled to −15 °C under Ar. To the cold reaction mixture was added NIS (175 mg, 0.77 mmol) followed by HClO_4_-SiO_2_ (30 mg) and it was stirred at same temperature for 30 min. After complete consumption of compound **3** [TLC; hexane/EtOAc 2:1], the reaction mixture was allowed to stir at 20 °C for 30 min and again cooled to −15 °C. To the cooled reaction mixture was added compound **3** (390 mg, 0.77 mmol) followed by NIS (175 mg, 0.77 mmol) and it was stirred at same temperature for 30 min. After complete consumption of compound **3** [TLC; hexane/EtOAc 2:1], the reaction mixture was allowed to stir at 20 °C for 30 min and again cooled to −15 °C. To the cooled reaction mixture was added compound **4** (350 mg, 0.81 mmol) followed by NIS (185 mg, 0.82 mmol) and it was stirred at the same temperature for 30 min. Then, the reaction mixture was filtered and washed with CH_2_Cl_2_ (30 mL). The organic layer was successively washed with 5% Na_2_S_2_O_3_ (25 mL), satd. NaHCO_3_ (25 mL), and H_2_O (25 mL), dried (Na_2_SO_4_) and concentrated to give the crude product, which was purified on SiO_2_ using hexane/EtOAc 2:1 as eluent to give pure compound **7** (320 mg, overall yield 30%).

## Supporting Information

File 1 Analytical data and NMR spectra of compounds **1**, **5**, **6** and **7**.
