# Supplementary material for: Synthesis of the tetrasaccharide repeating unit of the O-specific polysaccharide of Azospirillum doebereinerae type strain GSF71T using linear and one-pot iterative glycosylations
Source: Beilstein J Org Chem. 2020 Jul 15;16:1700–5. doi: 10.3762/bjoc.16.141 (PMC7372237; doi:10.3762/bjoc.16.141)
Supplement: File 1 — Analytical data and NMR spectra of compounds 1, 5, 6 and 7. [file Beilstein_J_Org_Chem-16-1700-s001.pdf]

BEILSTEIN JOURNAL OF ORGANIC CHEMISTRY

# Supporting Information

for

# Synthesis of the tetrasaccharide repeating unit of the *O*-specific polysaccharide of *Azospirillum doebereinerae* type strain GSF71$^T$ using linear and one-pot iterative glycosylations

Arin Gucchait, Pradip Shit and Anup Kumar Misra

*Beilstein J. Org. Chem.* **2020,** *16,* 1700–1705. doi:10.3762/bjoc.16.141

## Analytical data and NMR spectra of compounds 1, 5, 6 and 7

License and Terms: This is a supporting information file under the terms of the Creative Commons Attribution License (http://creativecommons.org/licenses/by/4.0). Please note that the reuse, redistribution and reproduction in particular requires that the authors and source are credited.
The license is subject to the *Beilstein Journal of Organic Chemistry* terms and conditions: (https://www.beilstein-journals.org/bjoc)

# Table of contents

**Subject** **page no.**

Analytical data of compounds **1, 5, 6** and **7** ........ S2–S4

1D ($^{1}$H and $^{13}$C NMR) and 2D NMR (COSY and HSQC) spectra of compounds **1**, **5**, **6** and **7** ........ S5–S12

## Analytical data of the synthesized products

***p*-Methoxyphenyl (2,4-di-*O*-benzyl-α-L-rhamnopyranosyl)-(1→3)-2-azido-4,6-*O*-benzylidene-2-deoxy-β-D-glucopyranoside (5):** Colorless syrup; $[\alpha]_D$ −11 (*c* 1.0, $CHCl_3$); $^1H$ NMR (500 MHz, $CDCl_3$): δ 7.42-6.80 (m, 19 H, Ar-*H*), 5.47 (s, 1 H, PhC*H*), 5.13 (br s, 1 H, H-$1_B$), 4.81 (d, *J* = 7.5 Hz, 1 H, H-$1_A$), 4.80 (d, *J* = 11.0 Hz, 1 H, PhC*H*), 4.74 (d, *J* = 11.0Hz, 1 H, PhC*H*), 4.62 (d, *J* = 11.5 Hz, 1 H, PhC*H*), 4.55 (d, *J* = 11.5 Hz, 1 H, PhC*H*), 4.35 (dd, *J* = 12.0 Hz, 5.5 Hz, 1 H, H-$6_{aA}$), 3.89-3.79 (m, 2 H, H-$3_B$, H-$5_B$), 3.77-3.74 (m, 5 H, H-$2_B$, H-$6_{bA}$, $OCH_3$), 3.66 (t, *J* = 9.5 Hz, 1 H, H-$3_A$), 3.60 (t, *J* = 8.0 Hz, 1 H, H-$2_A$), 3.55 (t, *J* = 9.0 Hz, 1 H, H-$4_A$), 3.49-3.46 (m, 1 H, H-$5_A$), 3.23 (t, *J* = 9.5 Hz, 1 H, H-$4_B$), 2.20-2.18 (m, 1 H, O*H*), 0.81 (d, *J* = 6.0 Hz, 1 H, $CCH_3$); $^{13}C$ NMR (125 MHz, $CDCl_3$): δ 155.9-114.6 (Ar-*C*), 102.1 (C-$1_A$), 102.0 (Ph*C*H), 97.8 (C-$1_B$), 82.2 (C-$4_B$), 79.1 (C-$4_A$), 77.9 (C-$2_B$), 75.8 (C-$3_A$), 74.6 ($PhCH_2$), 72.8 ($PhCH_2$), 71.2 (C-$3_B$), 68.4 (C-$6_A$), 67.4 (C-$5_B$), 67.0 (C-$2_A$), 66.8 (C-$5_A$), 55.4 ($OCH_3$), 17.3 ($CCH_3$); HRMS (ESI) calcd. for $C_{40}H_{43}N_3O_{10}$ (725.2948): $[M+H]^+$ 726.3026; found: 726.3034.

***p*-Methoxyphenyl (2,4-di-*O*-benzyl-α-L-rhamnopyranosyl)-(1→3)-(2,4-di-*O*-benzyl-α-L-rhamnopyranosyl)-(1→3)-2-azido-4,6-*O*-benzylidene-2-deoxy-β-D-glucopyranoside (6):** Colorless syrup; $[\alpha]_D$ −7 (*c* 1.0, $CHCl_3$); $^1H$ NMR (500 MHz, $CDCl_3$): δ 7.47-6.82 (m, 29 H, Ar-*H*), 5.54 (s, 1 H, PhC*H*), 5.17 (br s, 1 H, H-$1_C$), 5.11 (br s, 1 H, H-$1_B$), 4.93 (d, *J* = 11.5 Hz, 1 H, PhC*H*), 4.82 (d, *J* = 8.0 Hz, 1 H, H-$1_A$), 4.74-4.72 (m, 3 H, 3 PhC*H*), 4.65 (m, 3 H, 3 PhC*H*), 4.38 (dd, *J* = 10.5 Hz, 4.5 Hz, 1 H, H-$6_{aA}$), 4.07 (dd, *J* = 10.0 Hz, 3.0 Hz, 1 H, H-$3_B$), 4.04 (d, *J* = 11.5 Hz, 1 H, PhC*H*), 3.98-3.94 (m, 2 H, H-$5_B$, H-$6_{bA}$), 3.81 (t, *J* = 9.0 Hz, 1 H, H-$3_C$), 3.77 (s, 3 H, $OCH_3$), 3.73-3.71 (m, 2 H, H-$2_B$, H-$5_C$), 3.69-3.62 (m, 2 H, H-$2_C$, H-$3_A$), 3.60 (t, *J* = 9.0 Hz, 1 H, H-$2_A$), 3.56 (t, *J* = 9.5 Hz, 1 H, H-$4_A$), 3.54 (t, *J* = 9.5 Hz, 1 H, H-$4_C$), 3.50-3.45 (m, 1 H, H-$5_A$), 3.28 (t, *J* = 9.5 Hz, 1 H, H-$4_B$), 1.27 (d, *J* = 6.0 Hz, 3 H, $CCH_3$), 0.82 (d, *J* = 6.0 Hz, 3 H, $CCH_3$); $^{13}C$ NMR (125 MHz, $CDCl_3$): δ 129.0-114.6 (Ar-*C*), 102.1 (C-$1_A$), 102.0 (Ph*C*H), 98.7 (C-$1_C$), 98.4 (C-$1_B$), 82.2 (C-$4_B$), 80.9 (C-$4_C$), 79.2 (C-$2_C$), 79.0 (C-$4_A$), 77.4 (C-$3_B$), 76.9 (C-$2_B$), 75.8 (C-$3_A$), 74.9 ($PhCH_2$), 74.4 ($PhCH_2$), 72.5 ($PhCH_2$), 72.3 ($PhCH_2$), 71.6 (C-$5_B$), 68.5 (C-$6_A$), 68.4 (C-$3_C$), 67.7 (C-$2_A$), 67.0 (C-$5_C$), 66.8 (C-$5_A$), 55.5 ($OCH_3$), 18.0 ($CCH_3$), 17.2 ($CCH_3$); HRMS (ESI) calcd. for $C_{60}H_{65}N_3O_{14}$ (1051.4467): $[M+H]^+$ 1052.4545; found: 1052.4552.

***p*-Methoxyphenyl (2-*O*-acetyl-3,4-di-*O*-benzyl-α-L-rhamnopyranosyl)-(1→3)-(2,4-di-*O*-benzyl-α-L-rhamnopyranosyl)-(1→3)-(2,4-di-*O*-benzyl-α-L-rhamnopyranosyl)-(1→3)-2-azido-4,6-*O*-benzylidene-2-deoxy-β-D-glucopyranoside (7):** Colorless syrup; $[\alpha]_D$ − 12 (*c* 1.0, $CHCl_3$); $^1$H NMR (500 MHz, $CDCl_3$): δ 7.44-6.80 (m, 39 H, Ar-*H*), 5.51 (s, 1 H, PhC*H*), 5.46 (s, 1 H, H-$2_D$), 5.12 (br s, 1 H, H-$1_D$), 5.08 (br s, 1 H, H-$1_C$), 5.07 (br s, 1 H, H-$1_B$), 4.90 (d, *J* = 11.0 Hz, 1 H, PhC*H*), 4.80 (d, *J* = 8.0 Hz, 1 H, H-$1_A$), 4.79 (d, *J* = 11.0 Hz, 1 H, PhC*H*), 4.71 (s, 1 H, PhC*H*), 4.69 (br s, 1 H, PhC*H*), 4.64-4.53 (m, 5 H, 5 PhC*H*), 4.40 (d, *J* = 11.5 Hz, 1 H, PhC*H*), 4.36 (dd, *J* = 11.5 Hz, 5.5 Hz, 1 H, H-$6_{aA}$), 4.22 (d, *J* = 11.0 Hz, 1 H, PhC*H*), 4.20 (d, *J* = 11.0 Hz, 1 H, PhC*H*), 4.13 (dd, *J* = 9.5 Hz, 3.0 Hz, 1 H, H-$3_B$), 4.03 (dd, *J* = 10.0 Hz, 3.0 Hz, 1 H, H-$3_C$), 3.91-3.89 (m, 1 H, H-$5_B$), 3.89 (dd, *J* = 9.5 Hz, 3.0 Hz, 1 H, H-$3_D$), 3.83-3.77 (m, 2 H, H-$5_C$, H-$5_D$), 3.76 (s, 3 H, O$CH_3$), 3.67-3.65 (m, 3 H, H-$2_B$, H-$2_C$, H-$6_{bA}$), 3.67 (t, *J* = 9.0 Hz, 1 H, H-$3_A$), 3.60- 3.55 (m, H-$2_A$, H-$4_A$, H-$4_C$), 3.50 (t, *J* = 10.0 Hz, 1 H, H-$4_D$), 3.46-3.41 (m, 1 H, H-$5_A$), 3.37 (t, *J* = 9.0 Hz, 1 H, H-$4_B$), 2.08 (s, 3 H, COC$H_3$), 1.25 (d, *J* = 6.5 Hz, 3 H, CC$H_3$), 1.21 (d, *J* = 6.5 Hz, 3 H, CC$H_3$), 0.78 (d, *J* = 6.5 Hz, 3 H, CC$H_3$); $^{13}$C NMR (125 MHz, $CDCl_3$): δ 169.7 (*C*O$CH_3$), 155.9-114.6 (Ar-*C*), 102.1 (C-$1_A$), 101.9 (Ph*C*H), 99.3 (2 C, C-$1_B$, C-$1_C$), 98.4 (C-$1_D$), 80.9 (C-$4_C$), 80.8 (C-$4_D$), 79.9 (C-$4_B$), 79.0 (C-$4_A$), 78.5 (C-$2_B$), 77.8 (C-$3_D$), 77.7 (C-$3_C$), 77.4 (C-$3_B$), 77.1 (C-$2_C$), 75.9 (C-$3_A$), 75.1 (2 C, 2 Ph*C*$H_2$), 74.5 (Ph*C*$H_2$), 72.5 (Ph*C*$H_2$), 72.1 (Ph*C*$H_2$), 71.7 (Ph*C*$H_2$), 68.9 (C-$2_D$), 68.7 (C-$6_A$), 68.6 (C-$5_B$), 68.5 (C-$5_C$), 68.4 (C-$5_D$), 68.3 (C-$2_A$), 66.8 (C-$5_A$), 55.5 (O*C*$H_3$), 20.9 (CO*C*$H_3$), 18.2 (C*C*$H_3$), 18.0 (C*C*$H_3$), 17.2 (C*C*$H_3$); HRMS (ESI) calcd. for $C_{82}H_{89}N_3O_{19}$ (1419.6090): $[M+H]^+$ 1420.6168; found: 1420.6160.

***p*-Methoxyphenyl (2-*O*-acetyl-α-L-rhamnopyranosyl)-(1→3)-(α-L-rhamnopyranosyl)-(1→3)-(α-L-rhamnopyranosyl)-(1→3)-2-acetamido-6-*O*-acetyl-2-deoxy-β-D-glucopyranoside (1):** White powder; $[\alpha]_D$ −12 (*c* 1.0, $H_2O$); $^1$H NMR (500 MHz, $D_2O$): δ 6.96-6.88 (m, 4 H, Ar-*H*), 5.14 (d, *J* = 1.5 H, 1 H, H-$2_D$), 5.03 (br s, 1 H, H-$1_D$), 5.03 (d, *J* = 10.0 Hz, 1 H, H-$1_A$), 4.92 (br s, 1 H, H-$1_C$), 4.78 (br s, 1 H, H-$1_B$), 4.37 (d, *J* = 9.5 Hz, 1 H, H-$6_{aA}$), 4.30 (dd, *J* = 12.0 Hz, 5.5 Hz, 1 H, H-$6_{bA}$), 4.08 (s, 1 H, H-$3_C$), 4.01 (t, *J* = 9.0 Hz, 1 H, H-$2_A$), 3.99-3.95 (m, 2 H, H-$3_B$, H-$5_C$), 3.85-3.82 (m, 3 H, H-$2_B$, H-$3_D$, H-$5_B$), 3.76-3.72 (m, 6 H, H-$2_C$, H-$3_A$, H-$5_D$, OC$H_3$), 3.63-3.61 (m, 2 H, H-$4_A$, H-$5_A$), 3.52-3.41 (m, 3 H, H-$4_B$, H-$4_C$, H-$4_D$), 2.08 (OCOC$H_3$), 2.03 (OCOC$H_3$), 1.98 (NHCOC$H_3$), 1.26-1.24 (m, 6 H, 2 CC$H_3$), 1.23 (d, *J* = 6.0 Hz, 3 H, CC$H_3$); $^{13}$C NMR (125 MHz, $CDCl_3$): δ 174.3 (2 C, 2 O*C*O$CH_3$), 173.9 (NH*C*O$CH_3$), 119.1-99.3 (Ar-*C*), 102.2 (C-$1_C$), 101.3 (C-$1_B$), 99.8 (C-$1_A$), 99.3 (C-$1_D$), 81.2 (C-$4_A$), 78.4 (C-$2_B$), 78.1

(C-2$_C$), 73.5 (C-3$_A$), 72.5 (C-2$_D$), 72.2 (C-4$_D$), 71.2 (2 C, C-4$_B$, C-4$_C$), 70.4 (C-3$_D$), 69.8 (C-3$_B$), 69.2 (C-3$_C$), 69.1 (C-5$_D$), 69.0 (C-5$_C$), 68.5 (C-5$_B$), 68.3 (C-5$_A$), 63.1 (C-6$_A$), 55.7 (O*C*$H_3$), 55.1 (C-2$_A$), 22.0 (NHCO*C*$H_3$), 20.3 (OCO*C*$H_3$), 20.2 (OCO*C*$H_3$), 16.6 (C*C*$H_3$), 16.5 (C*C*$H_3$), 16.4 (C*C*$H_3$); HRMS (ESI) calcd. for $C_{37}H_{55}NO_{21}$ (849.3267): $[M+H]^+$ 850.3345; found: 850.3354.

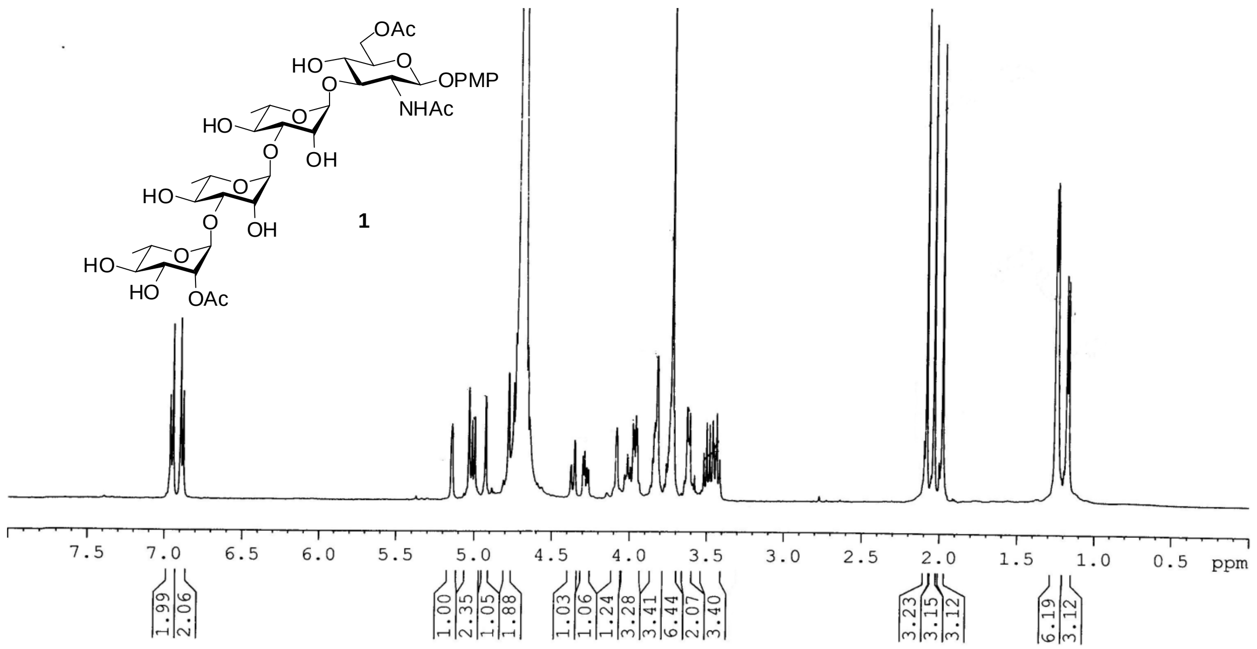

OAc
HO
O
HO
O
OPMP
NHAc
HO
O
OH
O
HO
O
OH
1
HO
O
HO
OAc
7.5
7.0
6.5
6.0
5.5
5.0
4.5
4.0
3.5
3.0
2.5
2.0
1.5
1.0
0.5
ppm
1.99
2.06
1.00
2.35
1.05
1.88
1.03
1.06
1.24
3.28
3.41
6.44
2.07
3.40
3.23
3.15
3.12
6.19
3.12


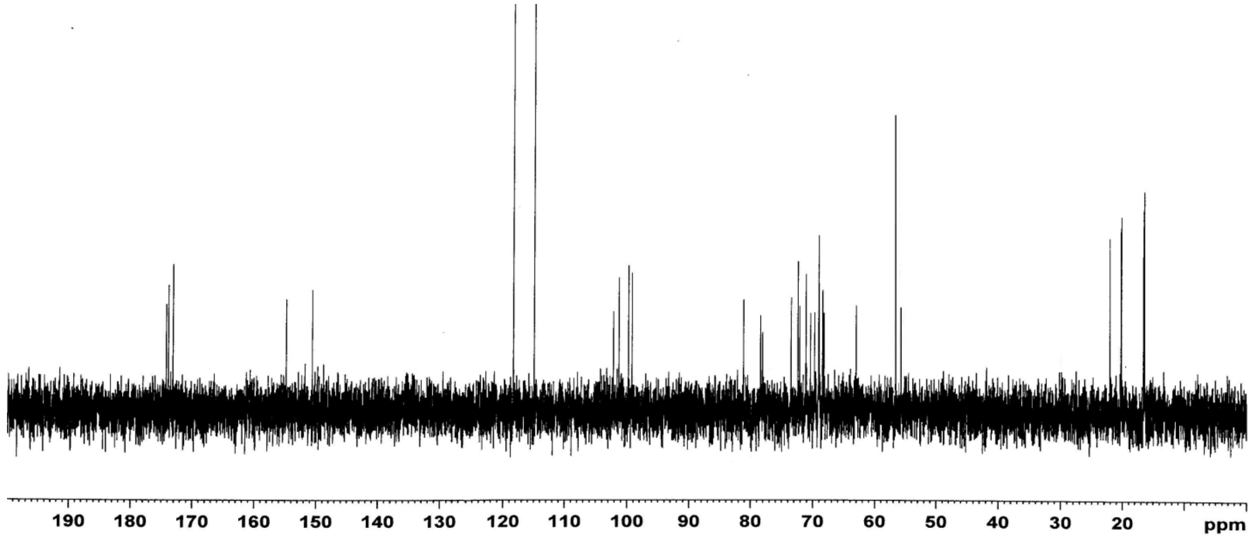

190 180 170 160 150 140 130 120 110 100 90 80 70 60 50 40 30 20 ppm


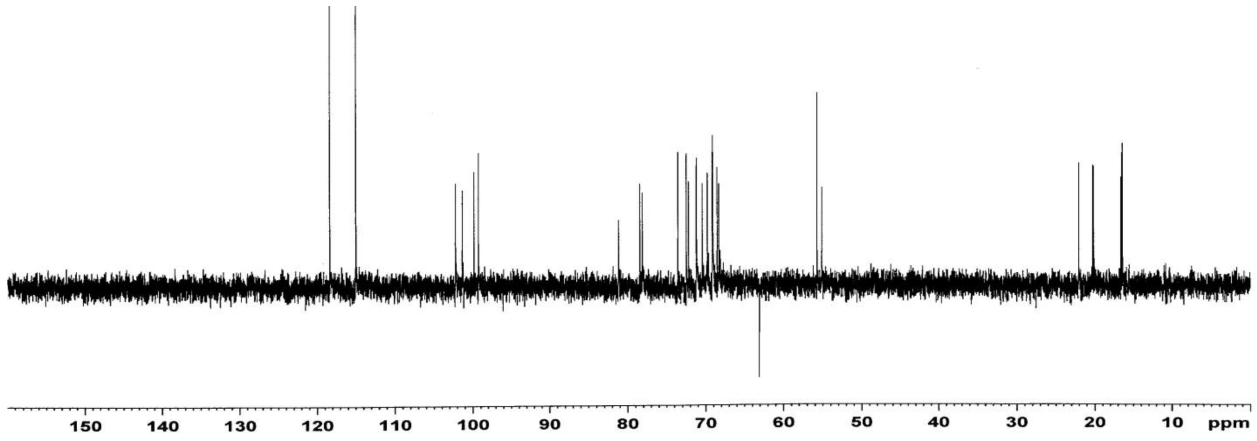

150 140 130 120 110 100 90 80 70 60 50 40 30 20 10 ppm

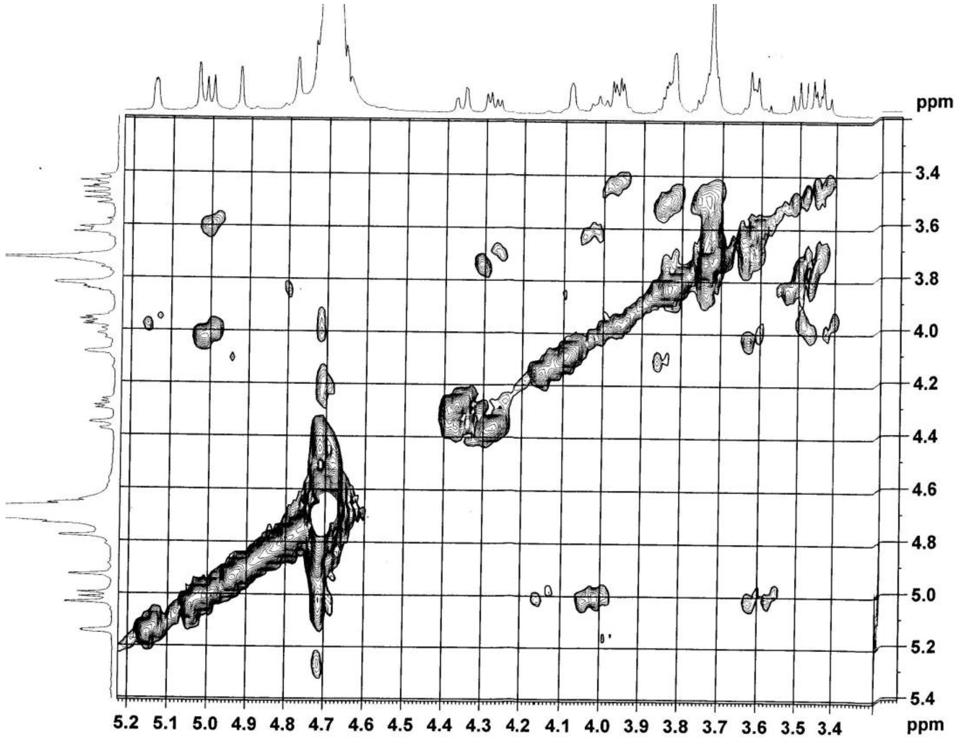
ppm
3.4
3.6
3.8
4.0
4.2
4.4
4.6
4.8
5.0
5.2
5.4
5.2 5.1 5.0 4.9 4.8 4.7 4.6 4.5 4.4 4.3 4.2 4.1 4.0 3.9 3.8 3.7 3.6 3.5 3.4
ppm

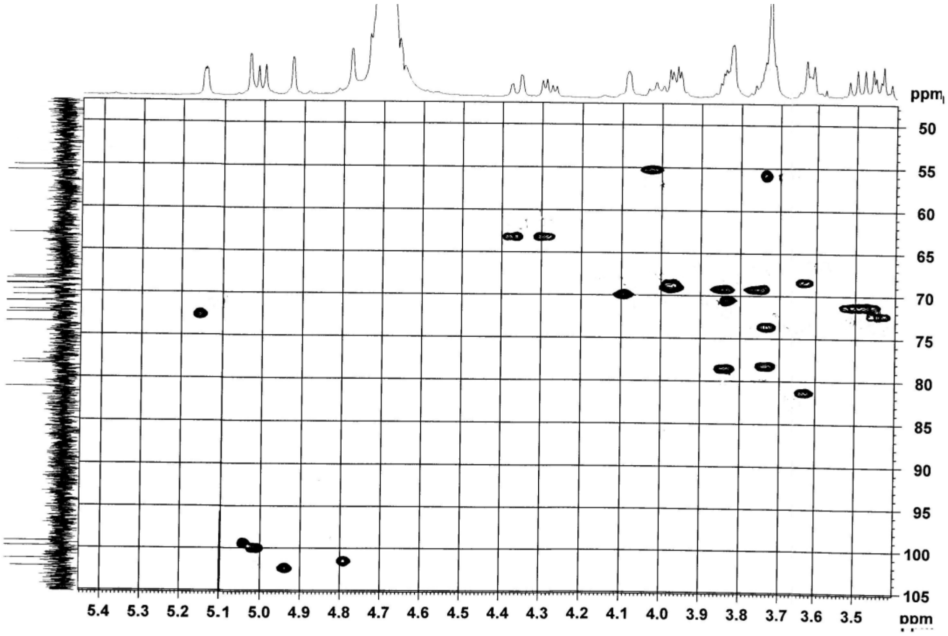
ppm
50
55
60
65
70
75
80
85
90
95
100
105
5.4 5.3 5.2 5.1 5.0 4.9 4.8 4.7 4.6 4.5 4.4 4.3 4.2 4.1 4.0 3.9 3.8 3.7 3.6 3.5
ppm

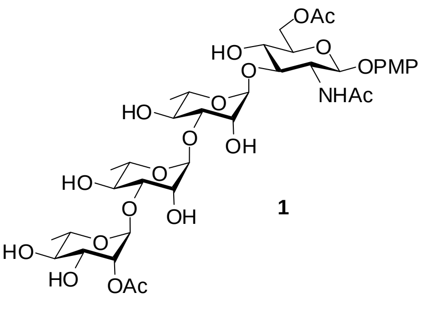
OAc
HO
O
OPMP
NHAc
HO
O
OH
HO
O
OH
HO
HO
OAc

**1**

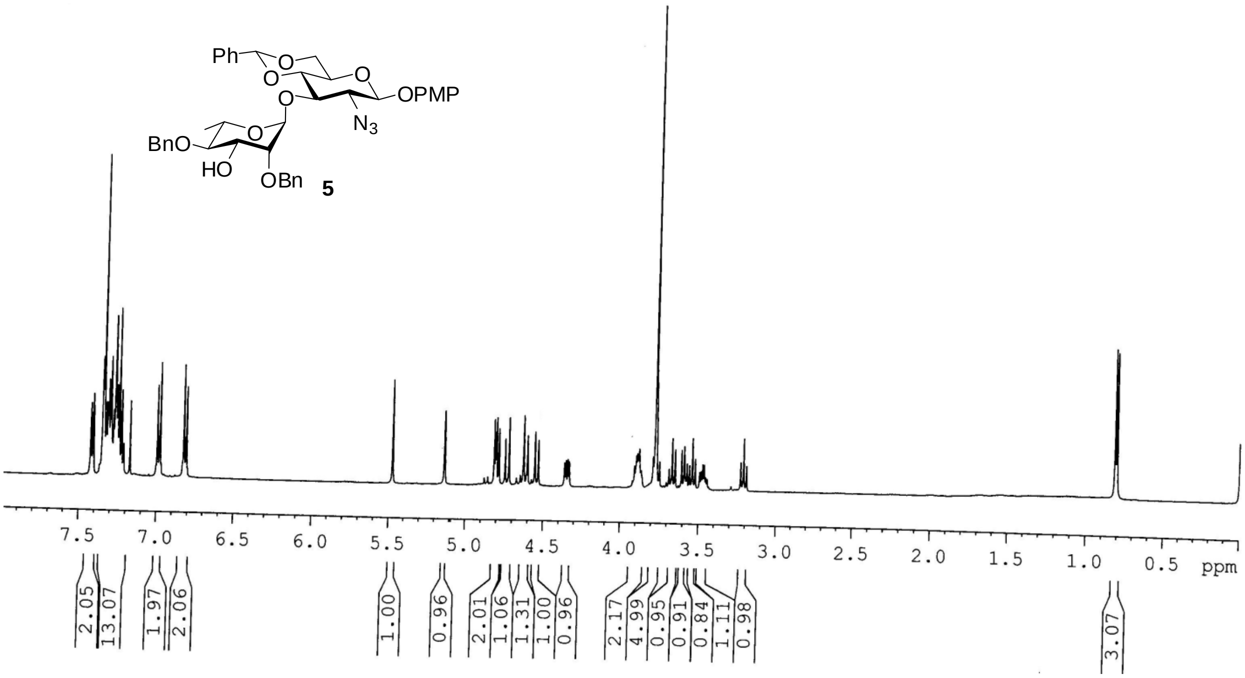
Ph
O
O
O
O
OPMP
$N_3$
BnO
O
HO
OBn
5
7.5 7.0 6.5 6.0 5.5 5.0 4.5 4.0 3.5 3.0 2.5 2.0 1.5 1.0 0.5 ppm
2.05
13.07
1.97
2.06
1.00
0.96
2.01
1.06
1.31
1.00
0.96
2.17
4.99
0.95
0.91
0.84
1.11
0.98
3.07

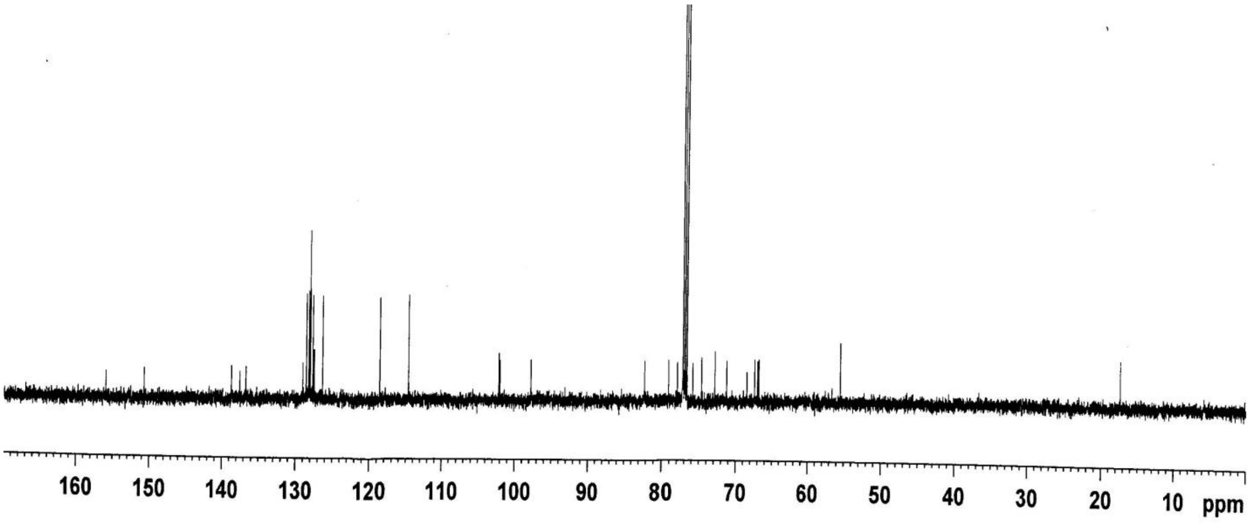
160 150 140 130 120 110 100 90 80 70 60 50 40 30 20 10 ppm

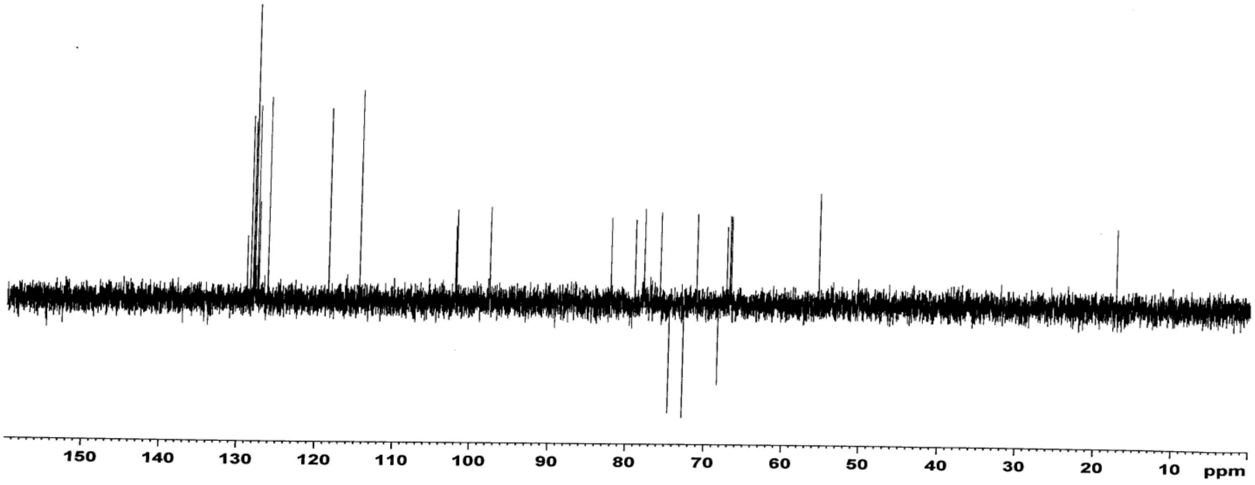
150 140 130 120 110 100 90 80 70 60 50 40 30 20 10 ppm

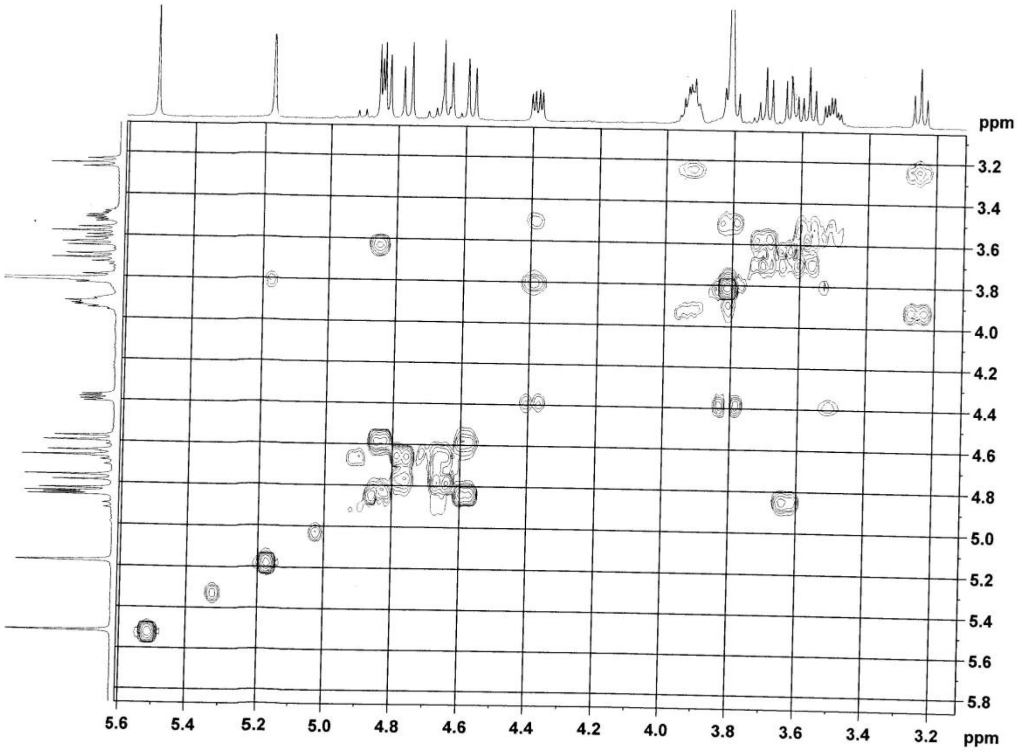

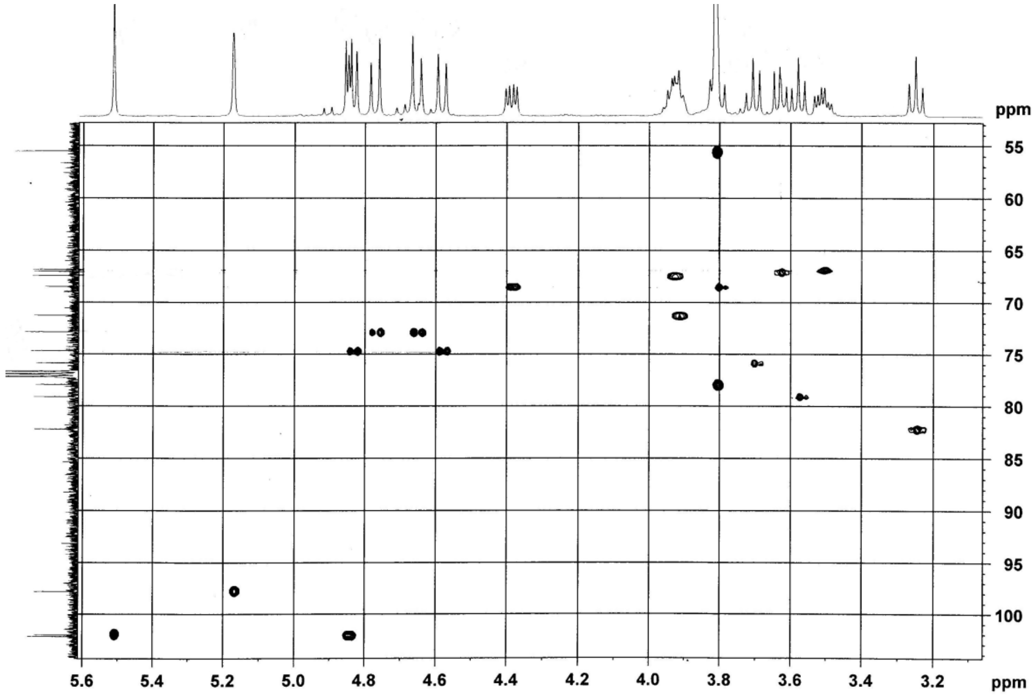

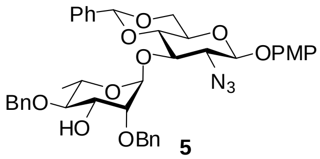


**5**

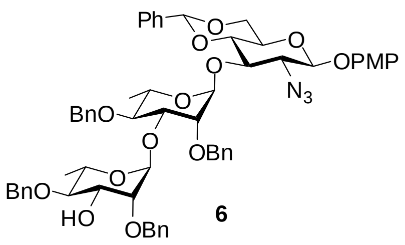
Ph
O
O
O
OPMP
N3
BnO
O
O
OBn
BnO
O
HO
OBn

**6**

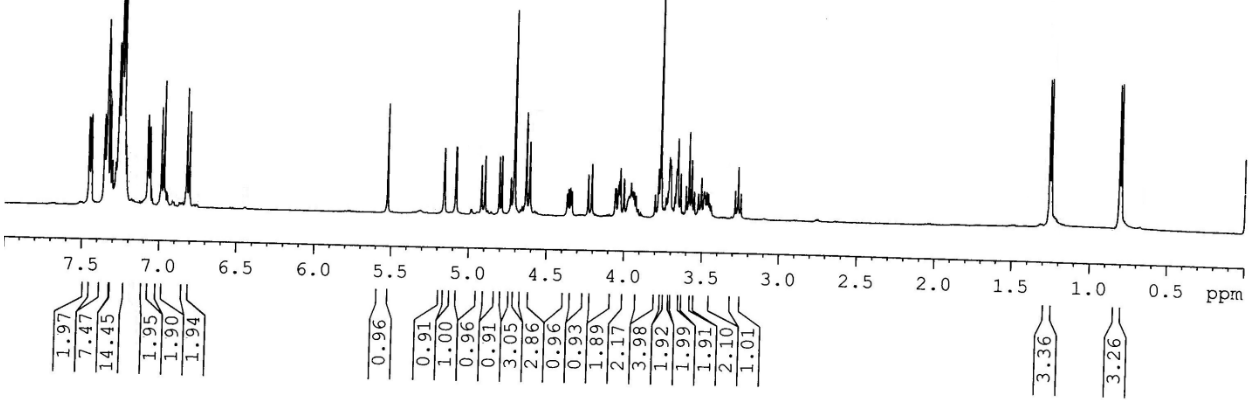
7.5 7.0 6.5 6.0 5.5 5.0 4.5 4.0 3.5 3.0 2.5 2.0 1.5 1.0 0.5 ppm
1.97 7.47 14.45 1.95 1.90 1.94 0.96 0.91 1.00 0.96 0.91 3.05 2.86 0.96 0.93 1.89 2.17 3.98 1.92 1.99 1.91 2.10 1.01 3.36 3.26

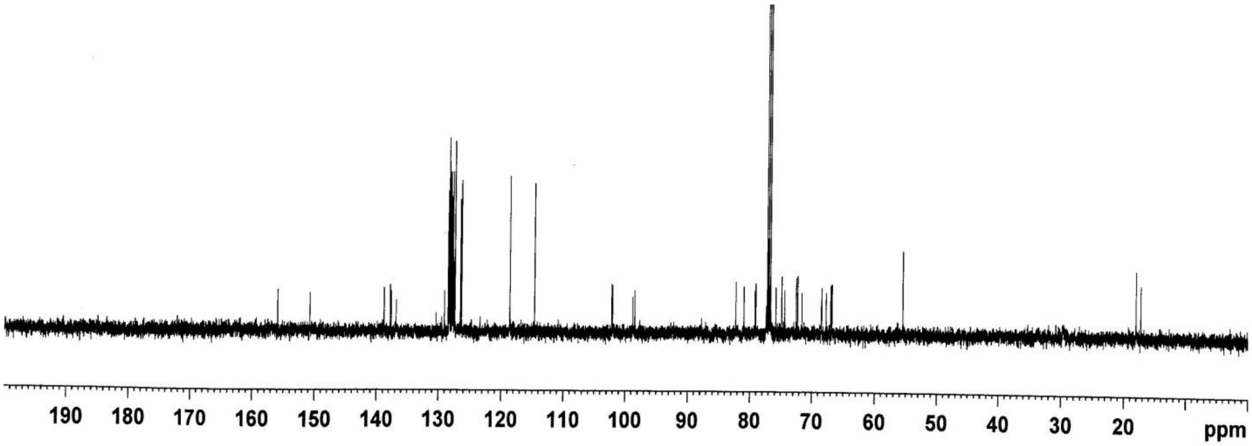
190 180 170 160 150 140 130 120 110 100 90 80 70 60 50 40 30 20 ppm

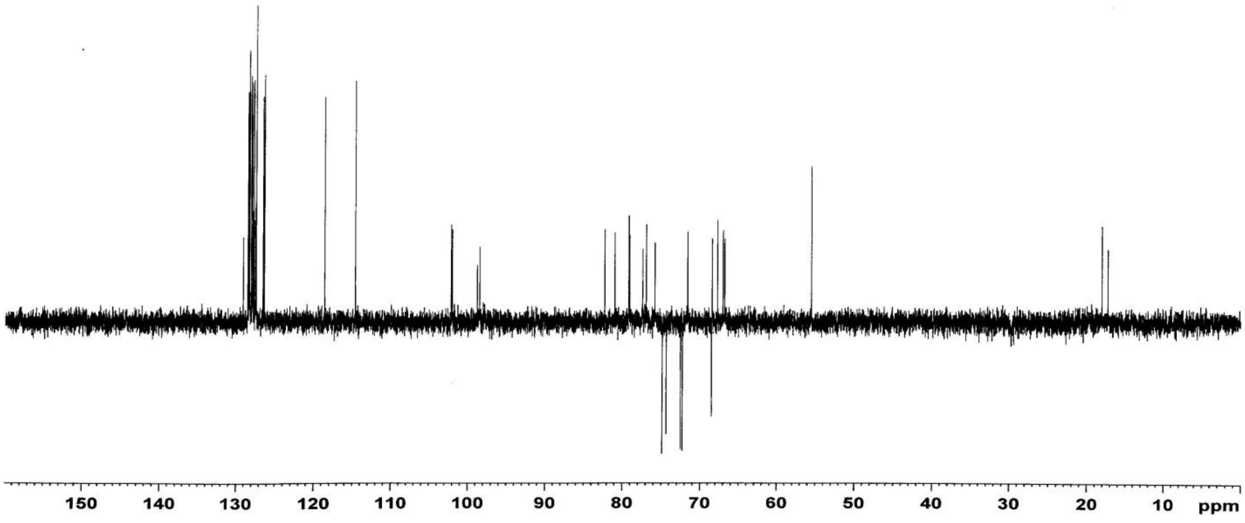
150 140 130 120 110 100 90 80 70 60 50 40 30 20 10 ppm

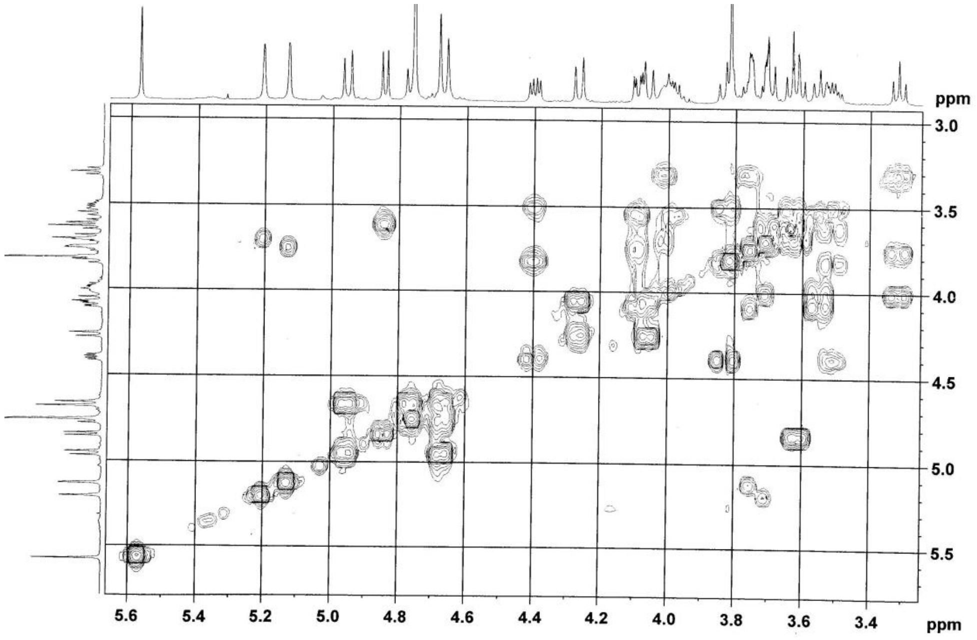
ppm
3.0
3.5
4.0
4.5
5.0
5.5
5.6 5.4 5.2 5.0 4.8 4.6 4.4 4.2 4.0 3.8 3.6 3.4
ppm

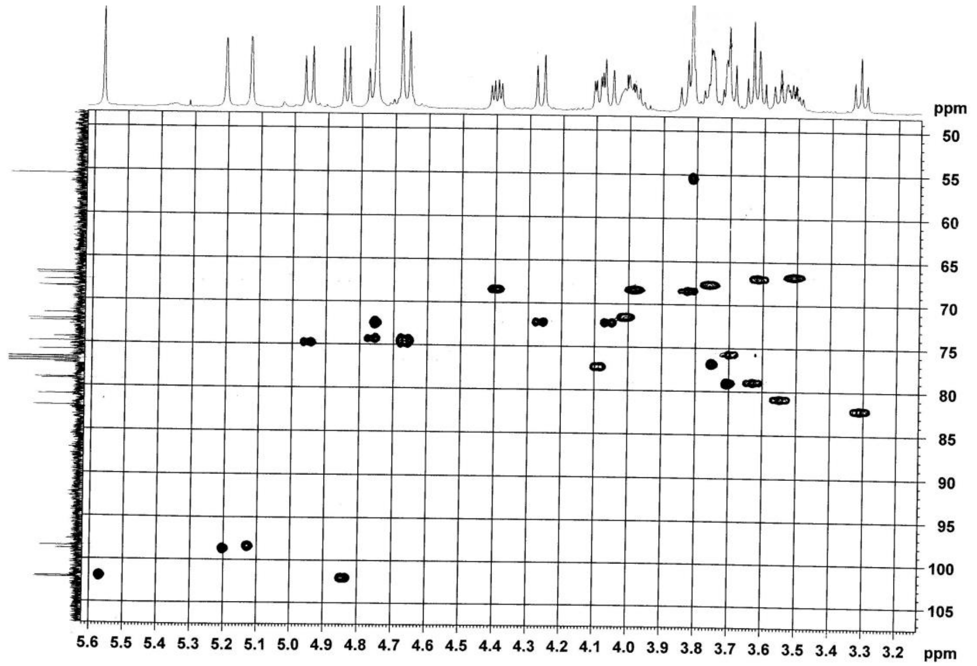
ppm
50
55
60
65
70
75
80
85
90
95
100
105
5.6 5.5 5.4 5.3 5.2 5.1 5.0 4.9 4.8 4.7 4.6 4.5 4.4 4.3 4.2 4.1 4.0 3.9 3.8 3.7 3.6 3.5 3.4 3.3 3.2
ppm

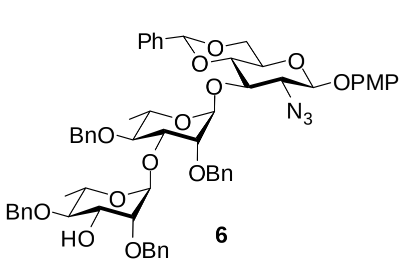
Ph
O
O
O
OPMP
$N_3$
O
BnO
O
OBn
O
O
BnO
HO
OBn

**6**

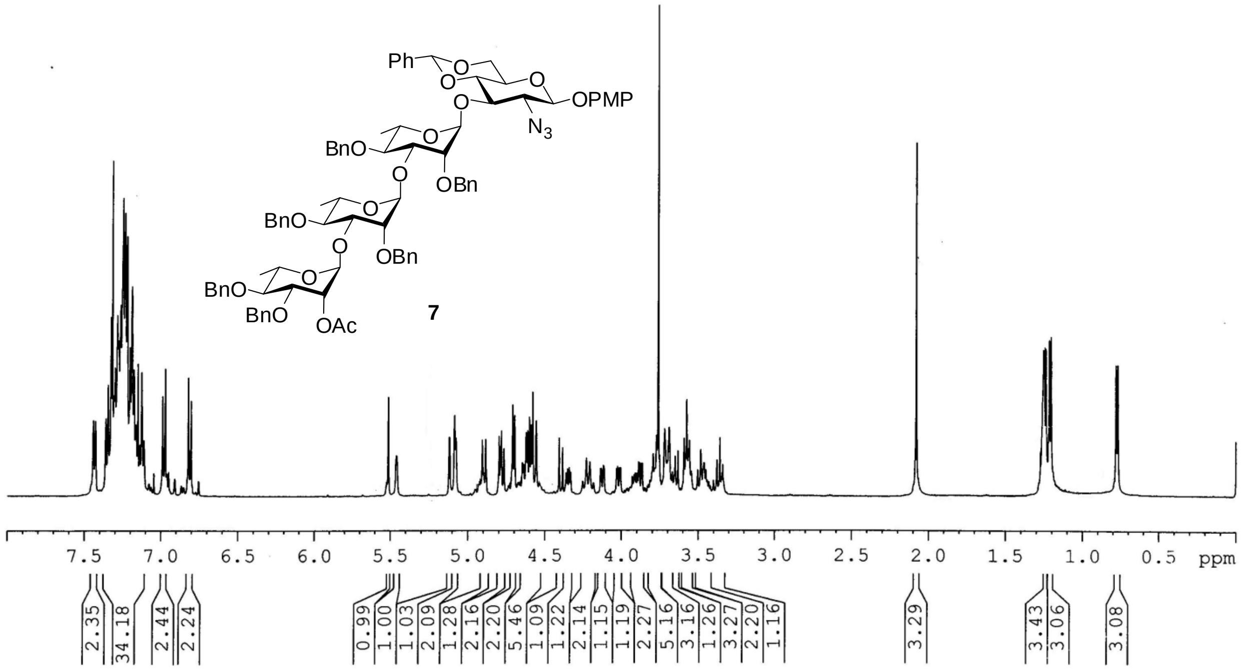
Ph
O
O
O
O
OPMP
$N_3$
BnO
O
OBn
BnO
O
O
OBn
BnO
O
BnO
OAc
7
7.5 7.0 6.5 6.0 5.5 5.0 4.5 4.0 3.5 3.0 2.5 2.0 1.5 1.0 0.5 ppm
2.35 34.18 2.44 2.24
0.99 1.00 1.03 2.09 1.28 2.16 2.20 5.46 1.09 1.22 2.14 1.15 1.19 2.27 5.16 3.16 1.26 3.27 2.20 1.16
3.29
3.43 3.06
3.08

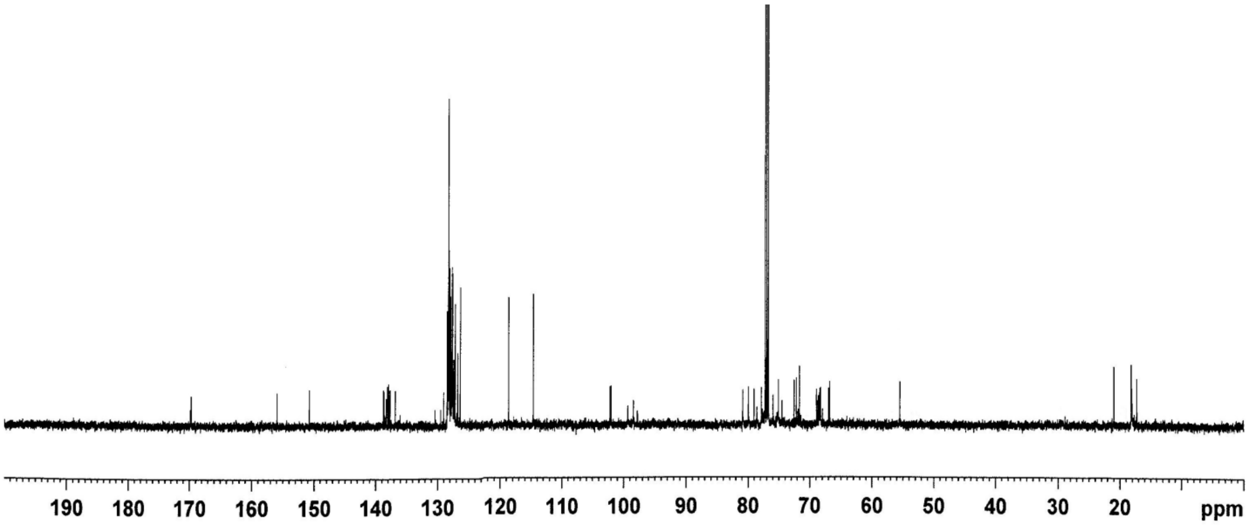
190 180 170 160 150 140 130 120 110 100 90 80 70 60 50 40 30 20 ppm

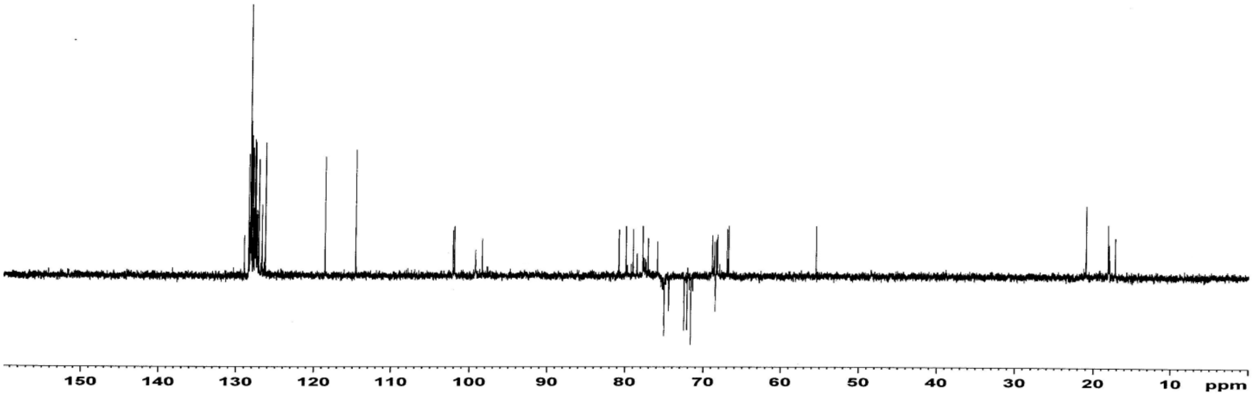
150 140 130 120 110 100 90 80 70 60 50 40 30 20 10 ppm

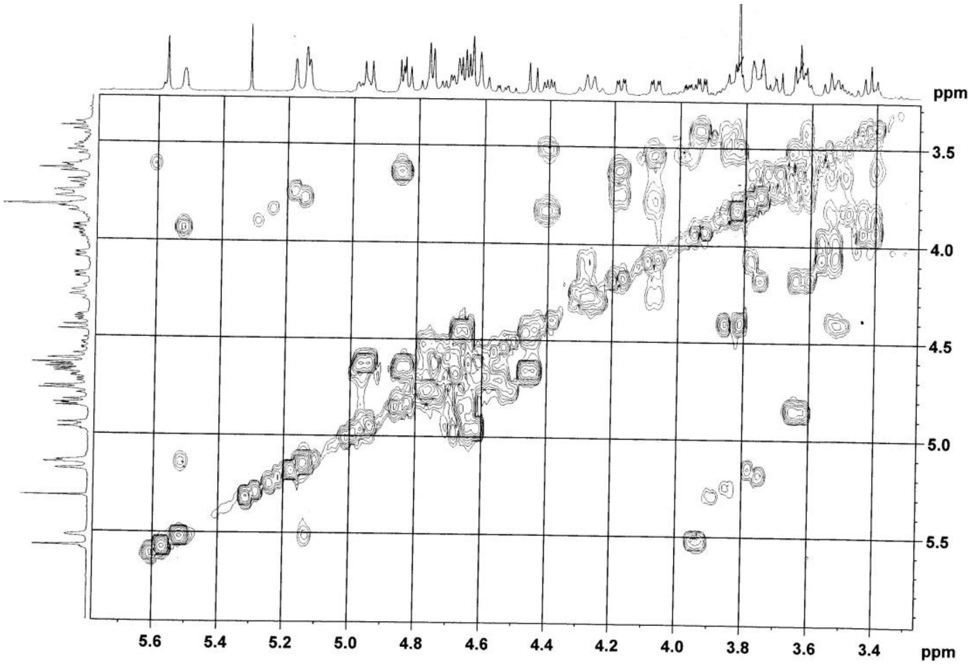
ppm
3.5
4.0
4.5
5.0
5.5
5.6 5.4 5.2 5.0 4.8 4.6 4.4 4.2 4.0 3.8 3.6 3.4
ppm

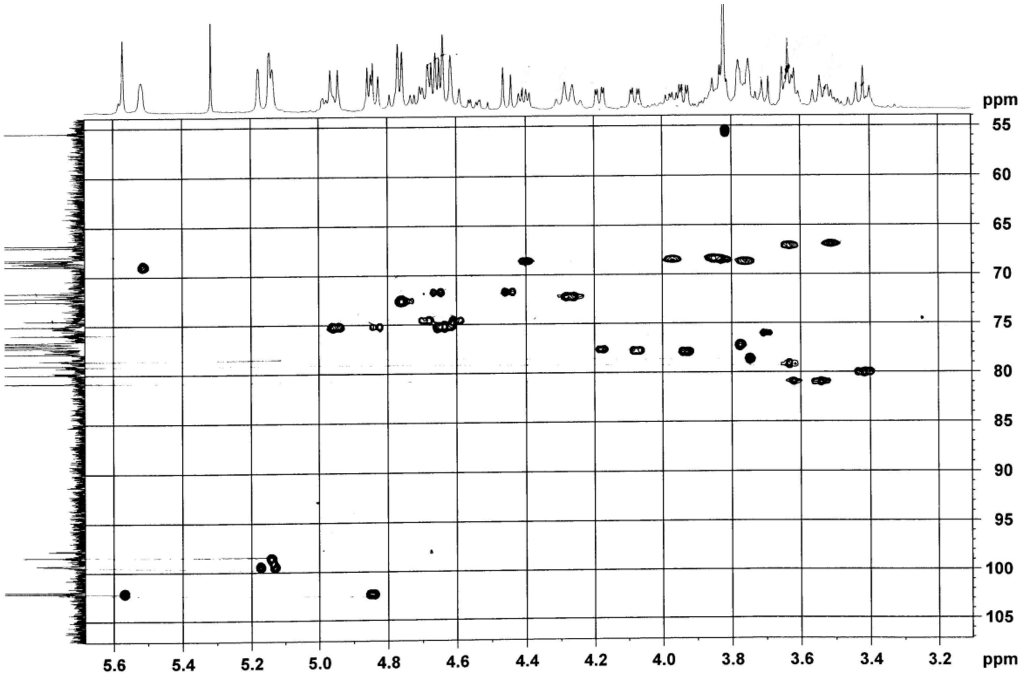
ppm
55
60
65
70
75
80
85
90
95
100
105
5.6 5.4 5.2 5.0 4.8 4.6 4.4 4.2 4.0 3.8 3.6 3.4 3.2
ppm

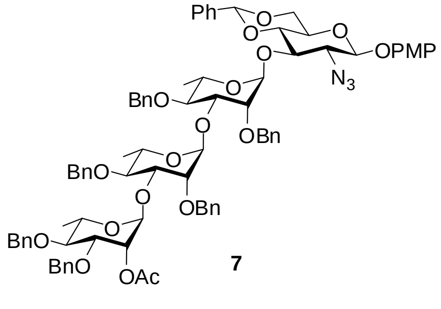
Ph
O
O
O
OPMP
O
$N_3$
BnO
O
OBn
O
BnO
O
O
OBn
BnO
O
BnO
OAc

**7**
